# Differential long-term outcomes for voluntary and involuntary transition from injection to oral opioid maintenance treatment

**DOI:** 10.1186/1747-597X-9-23

**Published:** 2014-06-08

**Authors:** Eugenia Oviedo-Joekes, Daphne Guh, Kirsten Marchand, David C Marsh, Kurt Lock, Suzanne Brissette, Aslam H Anis, Martin T Schechter

**Affiliations:** 1 Centre for Health Evaluation & Outcome Sciences, Providence Health Care, St. Paul’s Hospital, 575- 1081 Burrard St, Vancouver, BC V6Z 1Y6, Canada; 2 School of Population and Public Health, University of British Columbia, 2206 East Mall, Vancouver, BC V6T 1Z3, Canada; 3 Centre Hospitalier de l’Université de Montréal, Hôpital Saint-Luc, CHUM Montréal, Montréal, QC H2X 3 J4, Canada; 4 Centre for Addiction Research BC, University of Victoria, 2300 McKenzie Ave, Victoria, BC V8P 5C2, Canada; 5 Northern Ontario School of Medicine, 955 Oliver Road, Thunder Bay, ON P7B 5E1, Canada

**Keywords:** Opioid dependency, Diacetylmorphine, Injectable, Oral methadone, Opioid maintenance treatment

## Abstract

**Background:**

The most widely used maintenance treatment for opioid dependency is substitution with long-acting oral opioids. Treatment with injectable diacetylmorphine provides an opportunity for patients to stabilize and possibly transition to oral treatment, if clinically indicated. The aim of this study was to explore outcomes of individuals that received injectable diacetylmorphine and voluntarily transitioned to oral methadone.

**Design and methods:**

The North American Opiate Medication Initiative was a randomized controlled trial that compared the effectiveness of injectable diacetylmorphine (or hydromorphone) to oral methadone for long-term opioid-dependency. Treatment was provided for 12-months with an additional 3 months for transition and weaning. Participants were followed until 24-months from randomization. Among the participants randomized to injectable treatments, a sub-group voluntarily chose to transition to oral methadone (n = 16) during the treatment period. Illicit heroin use and treatment retention were assessed at 24-months for those voluntarily and involuntarily transitioning (n = 95) to oral methadone.

**Results:**

At 24-months, the group that voluntarily transitioned to oral methadone had higher odds of treatment retention (adjusted odds ratio = 5.55; 95% confidence interval [CI] = 1.11, 27.81; Chi-square = 4.33, df = 1, p-value = 0.037) than the involuntary transition group. At 24-months, the adjusted mean difference in prior 30 days of illicit heroin use for the voluntary, compared to the involuntary group was -5.58 (95% CI = -11.62, 0.47; t-value = -1.83, df = 97.4, p-value = 0.070).

**Conclusions:**

Although the results of this study were based on small groups of self-selected (i.e., non-randomized) participants, our data underlines the critical importance of voluntary and patient-centered decision making. If we had continued offering treatment with diacetylmorphine, those retained to injectable medication may have sustained the achieved improvements in the first 12 months. Diversified opioid treatment should be available so patients and physicians can flexibly choose the best treatment at the time.

**Trial registration:**

Clinical Trial Registration: NCT00175357

## Background

Dependency on heroin and other opioids is a chronic relapsing disease and continues to be a major public health concern. Individuals injecting street opioids are vulnerable to blood borne diseases, overdoses, premature death and involvement in criminal activities to sustain their drug use [1-3]. Currently, the most widely used and studied maintenance treatment for opioid dependency is substitution with methadone [4]. Methadone maintenance treatment (MMT) is effective at reducing the above harms associated with illicit opioid use [5]. For those for whom oral methadone is not effective, despite repeated attempts in the past, studies in Europe and Canada have demonstrated that medically supervised treatment with injectable diacetylmorphine (DAM) is effective [6,7]. Supervised injectable DAM is associated with increased retention in addiction treatment and decreased use of illicit substances, involvement in criminal activity and incarceration, and possibly reduced mortality [6,7]. Also, this treatment model provides an opportunity for individuals using illicit opioids to connect and engage with the addiction treatment system. Moreover, after starting treatment with DAM, some patients may choose to transfer to MMT. European studies suggest that among those who stopped treatment with injectable diacetylmorphine, approximately 30% per year transitioned to MMT [8,9]. The supervised model requires clinic visits two to three times per day, which has been suggested it could affect individuals’ psychosocial functioning [10]. Therefore, if clinically indicated and in consultation with the patient, transitions to other forms of treatments, in particular long acting oral opioids, might be a favourable outcome. The present study sought to determine outcomes of participants receiving injectable DAM in a Canadian trial who voluntarily transitioned to oral methadone during the study period compared to those whose treatment was discontinued as the study ended.

## Methods

### Study design, setting, participants and procedures

The North American Opiate Medication Initiative (NAOMI) study was a phase III, open-label randomized controlled trial that compared the effectiveness of injectable diacetylmorphine to oral methadone for treatment-refractory opioid-dependence. The study was conducted in Vancouver and Montreal, Canada, between March 2005 and July 2008. Study results, methodology and patients’ profiles have been published elsewhere [11-13]. Inclusion criteria were opioid dependence [14]; daily opioid injection; at least 5 years of opioid use; a minimum age of 25; a minimum of two previous treatments for opioid dependence including at least one attempt at MMT (in which 60 milligrams or more of methadone was received daily for at least 30 days within a 40 day period); and no enrolment in MMT within the prior 6 months. Participants were randomly assigned to receive oral methadone (n = 111), injectable diacetylmorphine (n = 115) or injectable hydromorphone (n = 25) for a period of 12 months. At the end of the 12-month study treatment period, participants’ still receiving injectable medications had three months to transition to other treatments available in the community (most likely oral methadone, as injectable medications were not available post-trial). All participants provided written informed consent. The trial received approval by the ethics review boards in each study site.

### Measures

Research evaluations were conducted at a separate research office by a team not involved in participants' treatment. This analysis presents data from the European Addiction Severity Index (EuropASI) [15], Maudsley Addiction Profile (MAP) [16] and EuroQol (EQ5D) [17], administered prior to randomization and at 12 and 24-months follow-up. A total of five participants withdrew their consent in the methadone group and one in the injectable diacetylmorphine group. The follow-up rates at 12 (end of treatment) and 24-months were 95.6% and 82.9%, respectively. To be considered retained to treatment, a participant must have been abstinent of opioids or confirmed to be receiving their study medication or any other addiction treatment during at least 10 of the 14 days prior to the 12-month assessment or 20 of the 30 days prior to the 24-month assessment.

### Statistical analyses

Data from participants randomized to either injectable diacetylmorphine or hydromorphone were combined, based on similar outcomes during the active treatment study period [18]. These participants were then divided into three groups: 1) ‘voluntary transition’ (n = 16) were those who voluntarily stopped receiving injectable medications and transitioned to oral methadone before the 12 month time-point; 2) ‘involuntary transition’ (n = 95) were those who were still receiving injectables at 12 months but were discontinued and offered other treatments because their study period ended; and 3) ‘withdrawal/drop outs’ (n = 28) were those who dropped out or were discontinued from the injectable medications before 12 months (various reasons: e.g., attempted to divert the study medications, violent behaviour, etc.). The primary outcomes for the present analyses were days of illicit heroin use and retention to treatment at 12 and 24-months follow-up.

Baseline characteristics were compared between the withdrawal, involuntary and voluntary transition groups using analysis of variance for continuous variables and chi-square tests for categorical variables. At 12 and 24-months follow-up, the overall mean differences for the EuropASI, MAP and EQ5D scores were compared using analysis of variance (F-test) and then pairwise comparisons were done using t-test for variables with the overall p-value of less than 0.05 in the analysis of variance. Multivariate regression models were also used to analyse each of the primary outcomes at 12 and 24- months for the voluntary and involuntary transition groups: 1) multivariate logistic regression models for retention; and 2) multivariate linear regression models for days of illicit heroin use. All regression models adjusted for baseline characteristics, including age, gender, ethnicity, chronic medical problems, years injecting drugs, prior 30 days of illicit heroin use, prior 30 days of illegal activities, and EuropASI-family sub-scale score. Finally, we also included an intention to treat analysis of the primary outcomes by the injectable and oral arms of NAOMI. There were no missing data at 24-months for retention. A multiple imputation technique was used for missing data and the adjusted degrees of freedom was used for inference [19]. Data were analyzed using SAS® (version 9.3) [20].

## Findings

Table 1 summarizes group characteristics at baseline. Compared to the involuntary transition group, there were less women and Aboriginal participants in the voluntary transition group. This group was also younger and had been injecting drugs for fewer years. However, statistically significant differences between groups were not observed for these variables, only for prior month days of illegal activities and the EuropASI Family sub-scale score reached statistical significance.

**Table 1:** Baseline characteristics for each transition group

**Variable at baseline**	**Injectable DAM or HDM (N = 139)**		
	**Withdraw/drop out (N = 28)**	**Involuntary transition (N = 95)**	**Voluntary transition (N = 16)**
Age^a^	39.7 ± 7.6	40.4 ± 7.9	37.6 ± 7.6
Female	9 (32.1%)	37 (38.9%)	4 (25.0%)
Aboriginal	9 (39.1%)	22 (29.3%)	1 (12.5%)
Chronic medical problem	19 (67.9%)	51 (53.7%)	7 (43.8%)
Previous MMT attempts	3.4 ± 1.7	3.1 ± 1.8	2.8 ± 1.0
Injecting drugs^a^	16.7 ± 7.9	16.9 ± 10.2	14.9 ± 8.0
Heroin use prior month^b^	27.7 ± 5.2	26.3 ± 8.2	26.4 ± 7.0
Cocaine use prior month^b^	20.8 ± 11.7	15.3 ± 12.6	14.9 ± 12.0
**Illegal activities prior month**^b, c^	**19.68 ± 11.43**	**14.60 ± 12.68**	**8.94 ± 11.66**
Injecting prior month^b^	16.71 ± 7.88	16.91 ± 10.23	14.88 ± 8.02
EuropASI-Drug^d^	0.57 ± 0.14	0.52 ± 0.14	0.55 ± 0.18
EuropASI-Legal^d^	0.45 ± 0.18	0.36 ± 0.26	0.27 ± 0.28
EuropASI-Medical^d^	0.43 ± 0.33	0.34 ± 0.35	0.41 ± 0.35
EuropASI-Econ^d^	0.88 ± 0.29	0.92 ± 0.23	0.83 ± 0.32
EuropASI-Job Satisfaction^d^	0.19 ± 0.26	0.26 ± 0.33	0.27 ± 0.34
**EuropASI-Family**^d, e^	**0.11 ± 0.14**	**0.08 ± 0.19**	**0.22 ± 0.26**
EuropASI-Social^d^	0.16 ± 0.22	0.10 ± 0.18	0.17 ± 0.23
EuropASI-Psychiatric^d^	0.24 ± 0.19	0.19 ± 0.21	0.26 ± 0.25
EQ5D (US weight)^f^	0.68 ± 0.21	0.72 ± 0.2	0.71 ± 0.22
EQ5D (UK weight)^g^	0.56 ± 0.31	0.62 ± 0.31	0.61 ± 0.34
MAP-Physical^h^	17.64 ± 7.54	14.74 ± 7.35	15.44 ± 9.65
MAP-Psychological^h^	15.5 ± 8.57	13.87 ± 7.78	14.81 ± 11.26

Numbers are: Mean ± standard deviation or N (%).

^(a)^Years.

^(b)^Days.

^(c)^Analysis of variance; F-test = 4.00, df = 2, 136, p-value = 0.021.

^(d)^EuropASI (European version of the Addiction Severity Index); sub-scale scores range from 0 to 1; higher scores are indicative of more severe problems.

^(e)^Analysis of variance; F-test = 3.73, df = 2, 136, p-value = 0.026.

^(f)^EQ5D (Euroquol) US weights: Scores range from -0.11 to 1; higher scores are indicative of better health status.

^(g)^EQ5D (Euroquol) UK weights: Scores range from -0.594 to 1; higher scores are indicative of better health status.

^(h)^MAP (Maudsley Addiction Profile). Scores range from 0 to 40; higher scores are indicative of more symptoms.

Table 2 shows retention and illicit heroin use for the voluntary and involuntary transition groups at 24 months. A higher proportion of voluntarily transitioning participants were retained at 24-months compared to the involuntary group. Results from the multivariate logistic regression model revealed that the voluntary transition group had 5.55 (95% CI = 1.11, 27.81; Chi-square = 4.33, df = 1, p-value = 0.037) times the adjusted odds of retention at 24-months compared to the involuntary transition group.

**Table 2:** Retention and illicit heroin use for voluntary compared to involuntary transition groups

**Outcome**	**Voluntary group**^**a **^**(n = 16)**	**Involuntary group**^**a **^**(n = 95)**	**Voluntary vs Involuntary**	**P Value**
			Adj. odds ratio (95% CI)	
Retention				
12 months	16 (100%)	95 (100%)	**-**	**-**
24 months^c^	14 (87.5%)	53 (55.8%)	5.55 (1.11, 27.81)	0.037^e^
			Adj. mean difference (95% CI)	
Illicit heroin use				
12 months^d^	5.66 (1.35, 9.96)	2.68 (1.45, 3.92)	4.02 (0.31, 7.73)	0.034^f^
24 months^c^	2.55 (0, 8.35)	10.61 (8.18, 13.04)	-5.58 (-11.62, 0.47)	0.070^g^

^(a)^Numbers are: Mean (95% Confidence Interval) or N (%).

^(b)^Models adjusted for baseline characteristics: age, gender, ethnicity, chronic medical problem, years injecting drugs, days of heroin use, days of illegal activities, European addiction severity index - family sub-scale score.

^(c)^Adjusted odds ratio from the logistic regression model; not tested at 12 months.

^(d)^Adjusted mean difference from the linear regression model.

^(e)^Wald chi-square test, chi-square value = 4.33, df = 1.

^(f)^t-test, t-value = 2.16, adjusted degrees of freedom for multiple imputation = 76.6.

^(g)^t-test, t-value = -1.83, adjusted degrees of freedom for multiple imputation = 97.4

**Figure 1:**
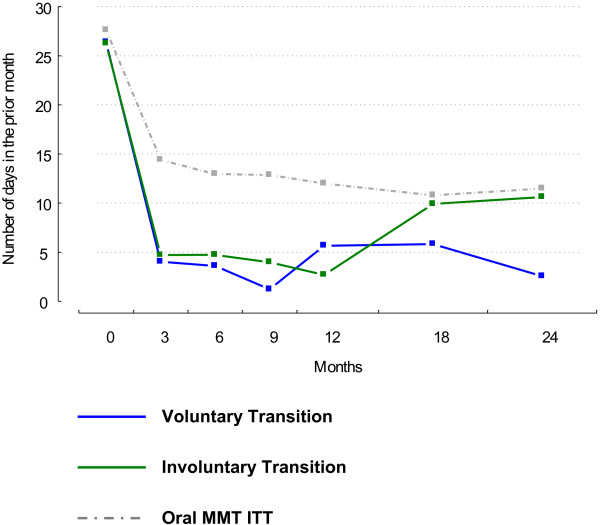
Days of illicit heroin use from baseline to 24-months follow-up for voluntary, involuntary and oral treatment groups.

Figure 1 illustrates the trajectory of illicit heroin use over the 24-month study period. All groups reduced their use of illicit heroin from baseline to 24-months. From Table 2 it is also evident that the mean prior 30 days of illicit heroin use was higher for the involuntary compared to the voluntary transition group at 24-months. The voluntary transition group had an unadjusted mean difference of 8.06 (95% CI = -14.35, -1.76; t-value = -2.53, adjusted df = 104.1, p-value = 0.013) days less of illicit heroin use in the prior 30 days than the involuntary transition group. The mean difference in days of illicit heroin use between the voluntary and involuntary transition groups at 24-months in the adjusted model was -5.58 days (95% CI = -11.62, 0.47; t-value = -1.83, df = 97.4, p-value = 0.07). Aside from illicit heroin use and retention to treatment, the voluntary and involuntary transition groups were similar in physical and psychological health and psychosocial outcomes at 24-months. Although, at 12-months, the voluntary transition group had a higher EuropASI- Social functioning score compared to the involuntary transition group (t-value = 2.14, adjusted df = 98.3, p-value = 0.034).

Table 3 displays ITT analysis of retention and illicit heroin use by randomization arm at 12 and 24-months. At 24 months the oral and injectable groups did not statistically differ in either retention or illicit heroin use. Days of illicit heroin use in the injectable group was 9.78 mean days (95% CI = 7.71, 11.85) and for those randomized to oral methadone was 11.48 mean days (95% CI = 9.1, 13.87). Retention rates were 77 (55.4%) and 60 (56.6%) for the injectable and oral group respectively.

**Table 3:** Retention and illicit heroin use by randomization arm

**Outcome**	**Injectable**^**c **^**(n = 139)**	**Oral **^**c **^**(n = 106)**	**Injectable vs Oral**	**P Value**
			Relative risk (95% CI)	
Retention^a^				
12 months	123 (88.5%)	60 (56.6%)	1.56 (1.31, 1.87)	<0.0001^d^
24 months	77 (55.4%)	60 (56.6%)	0.98 (0.78, 1.22)	0.851^e^
			Mean difference (95% CI)	
Illicit heroin use^b^				
12 months	5.23 (3.43, 7.04)	11.99 (9.91, 14.07)	-6.76 (-9.49, -4.02)	<0.0001^f^
24 months	9.78 (7.71, 11.85)	11.48 (9.1, 13.87)	-1.7 (-4.87, 1.47)	0.291^g^

All analysis are intention to treat.

^(a)^No missing data.

^(b)^With multiple imputation.

^(c)^Numbers are: Mean (95% Confidence Interval) or N (%).

^(d)^Chi-square test (chi-square value = 32.25, df = 1).

^(e)^Chi-square test (chi-square value = 0.04, df = 1).

^(f)^t-test, t-value = -4.87, adjusted degrees of freedom for multiple imputation = 240.6.

^(g)^t-test, t-value = -1.06, adjusted degrees of freedom for multiple imputation = 159.3.

## Discussion

The present analysis indicates that among individuals eligible for 12-months of treatment with injectable diacetylmorphine or hydromorphone in a clinical trial, a small sub-group of participants voluntarily transitioned from injection medication to oral methadone during the study. At the 24-month follow-up evaluation, these participants had higher retention to addiction treatment and fewer days of illicit heroin use in the prior 30 days, when compared to participants whose injection treatment was discontinued at 12-months due to the end of the clinical trial.

In NAOMI, the 12-month treatment endpoint was not mandated clinically but was due to the finite nature of the clinical trial. Individuals in NAOMI had an average of 16 years of prior heroin use, a history of MMT attempts and no MMT in the 6-months prior study inclusion, thus it is not surprising that the majority randomized to injectable medications remained on this treatment for the allocated treatment and transition period. It is noteworthy that a group of participants voluntarily transitioned to methadone, which they had previously rejected. It could be suggested that treatment with injectable diacetylmorphine provided them with an opportunity to stabilize and the participants opted to transition to oral methadone.

NAOMI was the only clinical trial with injectable diacetylmorphine that was unable to provide this treatment beyond the 12 month period due to logistical, financial and political reasons [12,21]. Despite differences in design and policies in place among studies, it is noteworthy that among those who discontinued treatment with diacetylmorphine in NAOMI (n = 44), voluntary transition rates to other treatments (primarily oral methadone) is similar to other contexts. For example, in the German study, among those who discontinued treatment, 36% of participants receiving treatment with diacetylmorphine voluntarily switched to other treatments, 27% to oral methadone and 9% to abstinence oriented treatment [8]. In the Swiss program, 2005 to 2010 yearly transition rates to oral methadone, as a reason to leave the program, have ranged from 35% to more than 45% among those discontinuing injectable diacetylmorphine [9]. The Swiss transition rates to other treatments described above do not account for those who were withdrawn or dropped out from diacetylmorphine treatment for other reasons (e.g., due to behavioral issues or moving away) and started MMT.

The involuntary transition group, despite adjusting for baseline differences and being retained for the 12-month treatment period, experienced an increase in their illicit heroin use at 24-months. Moreover, intention to treat analysis showed that although at 12 months the injectable arm had significantly higher retention and less illicit heroin use than the oral arm, at 24 months this difference disappeared. This evidence suggests that with continued provision of injectable medications, participants could have sustained the achieved positive outcomes and some voluntarily transition to transition to MMT, other treatments or abstinence. The German study, for example, found that some psychosocial outcomes might take as long as 4 years of treatment with injectable diacetylmorphine before reaching marked improvements [22].

Limitations of the NAOMI trial have been discussed elsewhere [11,12]. NAOMI was not designed to determine the outcomes of participants voluntarily transitioning to oral methadone; thus, the small sample sizes of the voluntary and involuntary transition groups impacts the statistical power of the present analysis and its conclusions. Motivating factors for transitioning from injectable treatments were not part of questionnaire data. Although, a qualitative sub-study [23] conducted with participants in both arms found that participants receiving injectable treatments were disappointed with the study ending. These participants discussed that the 12-month period was sufficient to experience many initial benefits but not long enough for those benefits to be fully sustained. Nevertheless, this study allows us to explore outcomes of those who voluntarily transitioned to oral MMT in the context of the Canadian trial.

These studies and our data underline the critical importance of patient-centered decision making. If injectable treatments were continued beyond the end of the study, some participants may have sustained the achieved improvements and some voluntarily transitioned to other alternatives in time. To be able to provide patient-centered care, an addiction treatment system should offer diversified opioid options for substitution treatment that would grant patients and doctors to choose the most effective treatment at a given time, case by case.

## Competing interests

The authors declare that they have no competing interests.

## Authors’ contributions

EOJ made substantial contributions to analysis, interpretation and lead preparation of manuscript; DG carried out analysis; KL made contributions to the acquisition of NAOMI data and drafting of the manuscript; KM was involved in revising the manuscript critically; DCM, SB, AA and MTS made substantial contributions to NAOMI’s conception and design and interpretation of the present data. All authors read and approved the final manuscript and agree to be accountable to the integrity of the work.
